# QuickStats

**Published:** 2014-05-09

**Authors:** FROM THE NATIONAL CENTER FOR HEALTH STATISTICS

**Figure:**
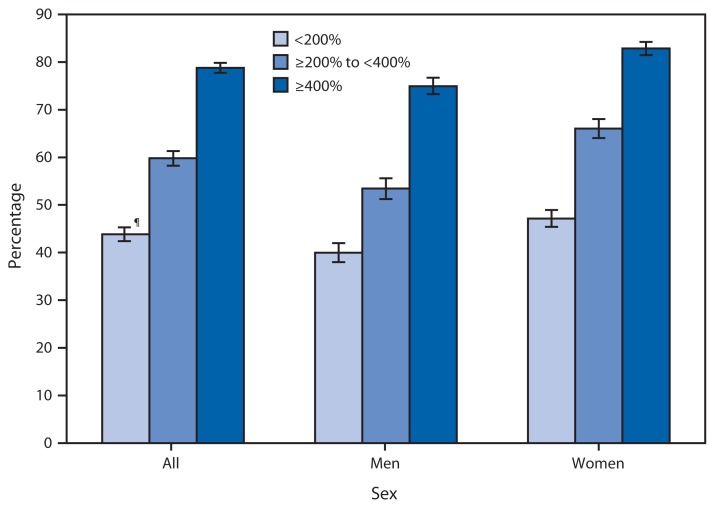
Percentage of Adults Aged 18–64 Years Who Have Seen a Dentist Within the Past Year,* by Family Income Group^†^ and Sex — National Health Interview Survey, United States, 2012^§^ * Based on response to the question, “About how long has it been since you last saw a dentist? Include all types of dentists, such as orthodontists, oral surgeons, and all other dental specialists, as well as dental hygienists.” ^†^ Family income groups were defined based on family income as a percentage of the federal poverty threshold. Poverty thresholds, which are published by the U.S. Census Bureau, vary by family size and the number of children in the family. Family income was imputed when missing using multiple imputation methodology. ^§^ Estimates are based on household interviews of a sample of the civilian, noninstitutionalized U.S. population and are derived from the National Health Interview Survey sample adult component. ^¶^ 95% confidence interval.

In 2012, the percentage of adults with a dental visit within the past year increased with increasing income. Approximately 44% of adults with family income <200% of the poverty threshold had a dental visit in the past year, increasing to 60% of those with family income from ≥200 to <400% and 79% for those with family income of ≥400% of the poverty threshold. The percentage of women with a dental visit in the past year was higher than men within each income group.

**Source:** National Health Interview Survey, 2012. Available at http://www.cdc.gov/nchs/nhis.htm.

**Reported by:** Brandy Lipton, PhD, blipton@cdc.gov, 301-458-4318; Sandra Decker, PhD.

